# The burden of treatment and compliance with the epinephrine auto-injector (EAI) in food allergic patients of all ages: a pilot study

**DOI:** 10.1186/2045-7022-1-S1-O44

**Published:** 2011-08-12

**Authors:** Jozephina C Kemna, Bertine MJ Flokstra-De Blok, Oude JNG Elberink, Anthony EJ Dubois

**Affiliations:** 1 University Medical Center Groningen, University of Groningen, Department of Paediatrics, Division of Paediatric Pulmonology and Paediatric Allergy, Groningen, Netherlands; 2 University Medical Center Groningen, University of Groningen, Department of General Practice, Groningen, Netherlands; 3 University Medical Center Groningen, University of Groningen, Department of Allergology, Groningen, Netherlands

## Background

Food allergic patients at risk for severe reactions should carry an epinephrine auto-injector (EAI). This treatment entails a burden for them and therefore may affect their compliance. Although the burden of treatment of an EAI has previously been shown to be high in vespid allergic patients, this has not yet been studied in food allergic patients. Therefore we determined the overall burden of treatment with the EAI in food allergic patients as perceived by both patients themselves and the parents of food allergic children. Furthermore, we examined the outcome of food allergic reactions requiring EAI use.

## Methods

Food allergic patients of all ages prescribed an EAI, attending an allergy clinic and the parents of food allergic children (aged 0-17 years) were asked to complete the following questionnaires: the Burden of Treatment instrument (BoT), which was our primary outcome, the Food Allergy Independent Measure questionnaire (FAIM), EAI positive and negative statements, questions concerning EAI prescription and use, and descriptive questions.

## Results

A total of 95 subjects were eligible for analysis. Two subjects were excluded because they did not complete the BoT. Of the remaining 93, 69 subjects (74%) were (extremely) positive about the EAI. Only 3 subjects were (slightly) negative about the EAI. In 31 patients epinephrine injection was needed, because of at least one allergic reaction. Out of these 31 patients, 11 did not carry an EAI with them at the time of the food allergic reaction. Only 2 patients (aged 16 and 21) were administered an EAI dose by someone other than themselves or their parents. There were no deaths.

## Conclusion

In contrast to vespid venom allergic patients, food allergic patients and their parents are (extremely) positive about carrying an EAI. Despite this, compliance with carrying the device or using it when necessary seems to be very low.

